# Laboratory Parameters in Detection of COVID-19 Patients with Positive RT-PCR; a Diagnostic Accuracy Study 

**Published:** 2020-04-04

**Authors:** Rajab Mardani, Abbas Ahmadi Vasmehjani, Fatemeh Zali, Alireza Gholami, Seyed Dawood Mousavi Nasab, Hooman Kaghazian, Mehdi Kaviani, Nayebali Ahmadi

**Affiliations:** 1 Department of Biochemistry, Pasteur Institute of Iran, Tehran, Iran.; 2 Department of Virology, School of Public Health, Tehran University of Medical Sciences, Tehran, Iran; 3 Department of Clinical Biochemistry, Faculty of Medicine, Tehran University of Medical Science, Tehran, Iran; 4 WHO Collaborating Center for Reference and Research on Rabies, Pasteur Institute of Iran, Tehran, Iran; 5 Department of Research and Development, Production and Research Complex, Pasteur Institute of Iran, Tehran, Iran; 6 Expert of Shohada-E Yaft Abad Hospital, Iran University of Medical Sciences, Tehran, Iran; 7 Proteomics Research Center, Shahid Beheshti University of Medical Sciences, Tehran, Iran

**Keywords:** SARS-CoV-2, COVID-19, Biomarkers, Biochemistry; blood cell count, Reverse Transcriptase Polymerase Chain Reaction

## Abstract

**Introduction:**

The role of laboratory parameters in screening of COVID-19 cases has not been definitely established. This study aimed to evaluate the accuracy of laboratory parameters in predicting cases with positive RT-PCR for COVID-19.

**Methods:**

This diagnostic accuracy study was conducted on suspected COVID-19 patients, who presented to Behpooyan Clinic Medical center in Tehran (Iran) from 22 February to 14 March, 2020. Patients were divided into two groups based on the results of real time reverse transcriptase‐polymerase chain reaction (RT-PCR) for COVID-19, and the accuracy of different laboratory parameters in predicting cases with positive RT-PCR was evaluated using area under the ROC curve (AUC).

**Results:**

Two hundred cases with the mean age of 41.3± 14.6 (range: 19-78) years were studied (0.53% male). The result of RT-PCR for COVID-19 was positive in 70 (35%) cases. Patients with positive RT-PCR had significantly higher neutrophil (NEU) count (p = 0.0001), and C-reactive protein (CRP) (p = 0.04), lactate dehydrogenase (LDH) (p = 0.0001), aspartate aminotransferase (AST) (p = 0.001), alanine aminotransferase (ALT) (p = 0.0001), and Urea (p = 0.001) levels in serum. In addition, patients with positive RT-PCR had lower white blood cell (WBC) count (p = 0.0001) and serum albumin level (p = 0.0001) compared to others. ALT (AUC = 0.879), CRP (AUC = 0.870), NEU (AUC = 0.858), LDH (AUC = 0.835), and Urea (AUC = 0.835) had very good accuracy in predicting cases with positive RT-PCR for COVID-19, respectively.

**Conclusion:**

Our findings suggest that level of LDH, CRP, ALT and NEU can be used to predict the result of COVID-19 test. They can help in detection of COVID-19 patients.

## Introduction

Compared to 2002/2003 SARS-CoV and 2012–2014 MERS-CoV epidemics, COVID-19 coronavirus rapidly spread to other parts of the world (185 countries and territories, Last updated: March 21, 2020)(1). 

In symptomatic patients, the clinical manifestations of the disease usually start after less than a week, consisting of fever (body temperature 37 to 38°C), cough, nasal congestion, and fatigue (2). Pneumonia mostly occurs in the second or third week of a symptomatic infection (3). Comparison of hematological parameters between mild and severe cases of COVID-19 showed significant differences in interleukin-6 (IL-6), D-Dimer, glucose (GLU), thrombin time (TT), fibrinogen (FIB) and C-reactive protein (CRP) (4). Fan et al. analyzed the hematological indices of COVID‐19 infected patients between the intensive care unit (ICU) and non‐ICU patients. They showed lymphopenia and raised lactate dehydrogenase (LDH) were associated with higher rate of ICU admissions. Patients who were transferred to the ICU had a lower nadir lymphocyte count, nadir monocyte count and nadir hemoglobin, and higher peak Neutrophil (NEU) Count and peak LDH levels compared to patients who did not require ICU stay (5) . Many patients with MERS-CoV had liver function abnormalities with elevated alanine aminotransferase (ALT), aspartate aminotransferase (AST), and LDH (6). Also laboratory data on SARS have shown that most patients had elevated CRP levels, lymphopenia, leukopenia, and elevated levels of aminotransferase, LDH and creatine kinase (7). A series of recently published articles have reported the epidemiological and clinical characteristics of patients with COVID-19 disease, but data regarding the laboratory characteristics of infected individuals are limited (8-10). This study aimed to evaluate the accuracy of laboratory parameters in predicting cases with positive RT-PCR for COVID-19.

## Methods

***Study design and setting***

This diagnostic accuracy study was conducted on suspected COVID-19 patients, who presented to Behpooyan Clinic Medical center in Tehran (Iran) from 22 February to 14 March, 2020. Patients were divided into two groups based on the results of real time reverse transcriptase‐polymerase chain reaction (RT-PCR) for COVID-19 and the accuracy of different laboratory parameters in predicting cases with positive RT-PCR was evaluated using area under the ROC curve (AUC). The study protocol was approved by the Ethics Committee of Shahid Beheshti University of Medical Sciences (ethical code: IR.SBMU.RETECH.REC.1399.010).

***Participants***

Outpatients with suspected COVID-19 having initial respiratory signs (including sore throat without shortness of breath), fever, cough, muscle ache, and headache were included (1). 

***Data gathering***

Pharyngeal swab samples were collected for COVID-19 test on presentation. Blood samples were collected from each participant and routine blood test including White blood cell count (WBC), Lymphocyte count (LYM), and Neutrophil count (NEU) were performed on the blood samples. Furthermore, blood biochemistry parameters such as Aspartate aminotransferase (AST), Alanine aminotransferase (ALT), Urea, C-reactive protein (CRP), as well as Albumin and lactate dehydrogenase (LDH) were assessed using HITACHI 7600-020 automated biochemistry analyzer.

**Statistical Analysis**

Data on Urea, WBC, Albumin, AST, ALT, LDH levels were expressed as mean ± standard deviation (SD). Differences in the levels of Urea, CRP, WBC, LYM, NEU, Albumin, AST, ALT and LDH between the RT-PCR positive and negative patients were assessed using student’s t-test. Receiver operating characteristic (ROC) curve and AUC were used to analyze the optimal cut-off for prediction of positive RT-PCR cases. In this study, AUC 0.9 to 1 was defined as excellent accuracy, 0.8 to 0.9 as very good, 0.7 to 0.8 as good, 0.6 to 0.7 as sufficient, 0.5 to 0.6 as bad, and < 0.5 as poor (useless test).

## Results

***Characteristics of the studied cases***

Two hundred cases with the mean age of 41.3± 14.6 (range: 19-78) years were studied (0.53% male). 40.2% of cases were in the 30 to 49 years age range. The result of RT-PCR for COVID-19 was positive in 70 (35%) cases and negative in 130 (65%). Groups of patients with positive and negative RT-PCR were similar regarding gender (p = 0.17) and age (p = 0.35) distribution.

***Laboratory parameters***

Table 1 compares the laboratory parameters of patients with positive and negative RT-PCR. Patients with positive RT-PCR had significantly higher NEU count (p = 0.0001), and CRP (p = 0.04), LDH (p = 0.0001), AST (p = 0.001), ALT (p = 0.0001), and Urea (p = 0.001) levels in serum. In addition, patients with positive RT-PCR had lower WBC count (p = 0.0001) and serum albumin level (p = 0.0001) compared to others. 

**Table 1:** Comparing the laboratory parameters between the cases with positive and negative RT-PCR for COVID-19 infection

**Parameters**	**Total (n=200)**	**RT-PCR for COVID-19**		***P***
		**Positive (n=70)**	**Negative (n=130)**	
WBC (cell/mm3)	5962.8±2127	4043±1002	6894±1982	0.0001
NEU (%)	51.9	60.7	47.8	0.0001
LYM (%)	46.7	37.7	51.8	0.0001
Positive CRP ^a^ (%)	37	54	27.6	0.04
AST (IU/L)	28.6±8.6	32.1±8.01	26.8±8.3	0.001
ALT (IU/L)	30±9.1	37.8±7.9	26.2±6.9	0.0001
LDH (U/L)	372.5±115	465.2±100.2	327.6±93.2	0.0001
Urea (mg/dl)	28.6±8.01	34.6±8.6	25.8±5.8	0.001
Albumin (g/dl)	3.5±0.9	2.9±0.8	3.7±0.8	0.0001

^a ^CRP test is qualitative and the indicated number shows the percentage of positive results in each group. Abbreviations: White blood cell count (WBC), Lymphocyte (LYM), Neutrophil (NEU), Aspartate aminotransferase (AST), Alanine aminotransferase (ALT), C-reactive protein (CRP), and lactate dehydrogenase (LDH).

**Table 2:** The area under the receiver operating characteristic (ROC) curve (AUC) of the studied parameters in predicting cases with positive RT-PCR for COVID-19

**Variables **	**Cut-off **	**AUC**	**95% CI**	***P***
White blood cell (cells/mm3)	0.6	0.075	0.03-0.11	0.09
Neutrophils (%)	0.70	0.858	0.79-0.92	<0.0001
Lymphocyte (%)	0.6	0.112	0.05-0.16	0.12
Positive C-reactive protein (%)	0.70	0.870	0.72-0.88	0.002
Aspartate aminotransferase (IU/L)	0.40	0.716	0.63-0.8	<0.0001
Alanine aminotransferase (IU/L)	0.40	0.879	0.82-0.93	<0.0001
lactate dehydrogenase (U/L)	0.70	0.835	0.76-0.9	<0.0001
Urea (mg/dl)	0.70	0.831	0.76-0.9	<0.0001
Albumin (g/dl)	0.6	0.242	0.15-0.32	0.04

CI: confidence interval.

**Figure 1:**
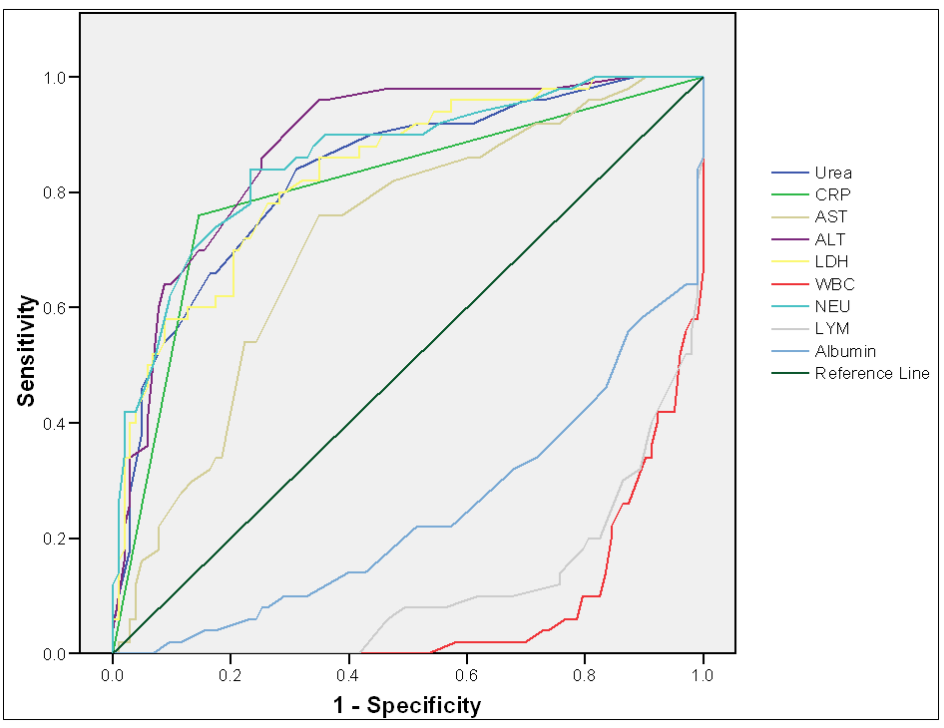
Area under the receiver operating characteristic curve of different laboratory parameters in predicting cases with positive RT-PCR for COVID-19.

Table 2 and figure 1 show the area under the ROC curve of studied parameters in predicting cases with positive RT-PCR for COVID-19. ALT (AUC = 0.879), CRP (AUC = 0.870), NEU (AUC = 0.858), LDH (AUC = 0.835), and Urea (0.835) had very good accuracy in predicting cases with positive RT-PCR for COVID-19, respectively.

## Discussion

Based on the findings of this study ALT, CRP, NEU, LDH, and Urea have very good accuracy in predicting cases with positive RT-PCR for COVID-19, respectively.

Chen et al., found that LDH had significantly increased in most patients, while albumin had decreased, but ALT and AST showed no significant changes (11). The mentioned values were also reported for patients with MERS-CoV, where elevated ALT, AST and LDH was observed (6). Another study indicated that 2–11% of patients with COVID-19 had liver comorbidities and 14–53% of cases had abnormal ALT and AST levels during progression of COVID-19 disease (12). Furthermore, Shi et al. studied patients whose COVID-19 diagnosis was confirmed by computed tomography (CT) scan while in the subclinical phase and found that incidence of AST abnormality among these patients was significantly lower than those diagnosed after the onset of symptoms (13). Therefore, liver injury is more prevalent in severe cases compared to mild cases of COVID-19. In another report, Yang et al. found no difference in the incidence of abnormal liver function between survivors (30%) and non-survivors (28%) (9). Liver damage in mild cases of COVID-19 is often transient and can return to normal without any special treatment (12).

We have found that the number and percentage of WBC, LYM and NEU were significantly different between positive and negative RT-PCR cases for COVID-19/or SARS-CoV-2. In comparison to the normal range, we found low WBC and LYM counts in patients with positive RT-PCR COVID-19, whereas NEU counts were higher in these patients. In previous reports, low LYM and WBC counts were found in most patients, which is in line with our study (14). Laboratory studies showed leucopenia with leukocyte counts of 2.91 × 10^9^ cells/L, 70.0% of which were NEU (15). Therefore, our result suggests that NEU might not be affected with SARS-CoV-2 in the initial phase of the disease. It also suggests that SARS-CoV-2 might mainly act on lymphocytes, especially T lymphocytes, as does SARS-CoV. Virus particles spread through the respiratory tract and infect other cells, inducing series of immune responses, and causing changes in number of peripheral white blood cells such as lymphocytes (11). Some studies suggest that a substantial decrease in the total number of lymphocytes indicates that coronavirus affects many immune cells and inhibits cellular immune function (11). Tsui and others reported that high neutrophil count on admission of COVID-19 patients, and elevated LDH level were independent predictors of an adverse clinical outcome (16).

In the present study, ROC curve was used to analyze the specificity and sensitivity of different variables in suspected COVID-19 patients. The AUC of laboratory parameters such as ALT, CRP, AST, LDH, and NEU indicated that they could be used to predict the presence of COVID-19 disease, while those of albumin and WBC were below the reference line of ROC curve, indicating that they were poor predictors of the disease. The data is in line with results reported by Wang et al. (17) and Gao et al. (4). In the current study, the AUC of CRP, ALT, LDH, urea and NEU were above 0.80; thus, they are effective and have very good predictive value for predicting COVID-19. It seems that, some blood laboratory parameters could be used in screening cases with positive RT-PCR for COVID-19.

Considering the significant difference in laboratory parameters evaluated in this study between the 2 groups, one can hope to model or predict the results of coronavirus testing based on routine laboratory tests. 

## Limitations

The sample size was relatively small. In addition, since this study was conducted on blood laboratory parameters, not every patient was continuously monitored for all clinical manifestations.

## Conclusion

Based on the findings of this study ALT, CRP, NEU, LDH, and Urea have very good accuracy in predicting cases with positive RT-PCR for COVID-19, respectively.
